# Airway Mucus Plugs in Community-Living Adults: A Study Protocol

**Published:** 2024-05-24

**Authors:** Maya Abdalla, Rim Elalami, Michael H Cho, George T O’Connor, Mary Rice, Michael Horowitz, Neda Akhoundi, Andrew Yen, Ravi Kalhan, Alejandro A. Diaz

**Affiliations:** 1 Division of Pulmonary and Critical Care, Department of Medicine, Brigham and Women’s Hospital, Boston, MA, USA; 2 Division of Pulmonary and Critical Care, Department of Medicine, Harvard Medical School, Boston, MA, USA; 3 Pulmonary Center, Department of Medicine, Boston University School of Medicine, Boston, Massachusetts, USA; 4 The National Heart, Lung, and Blood Institute’s Framingham Heart Study, Framingham, Massachusetts, USA; 5 Channing Division of Network Medicine, Department of Medicine, Brigham and Women’s Hospital, Harvard Medical School, Boston, MA, USA; 6 Division of Pulmonary, Sleep and Critical Care Medicine, Beth Israel Deaconess Medical Center, Boston, Massachusetts, USA; 7 Department of Radiology, University of California, San Diego, 9452 Medical Center Dr, 4th Floor, La Jolla, CA 92037; 8 Northwestern University Feinberg School of Medicine, 1700 W. Van Buren St, Ste. 470, 60612, Chicago, IL, USA

**Keywords:** Airway disease, Mucus pathology, Mucus plugs, Chronic Obstructive Pulmonary Disease (COPD), Lung imaging, Population-based study, Computed Tomography (CT)

## Abstract

**Introduction:**

Mucus pathology plays a critical role in airway diseases like Chronic Bronchitis (CB) and Chronic Obstructive Pulmonary Disease (COPD). Up to 32% of community-living persons report clinical manifestations of mucus pathology (e.g., cough and sputum production). However, airway mucus pathology has not been systematically studied in community-living individuals. In this study, we will use an objective, reproducible assessment of mucus pathology on chest Computed Tomography (CT) scans from community-living individuals participating in the Coronary Artery Risk Development in Young Adults (CARDIA) and Framingham Heart Study (FHS) cohorts.

**Methods and analysis:**

We will determine the clinical relevance of CT-based mucus plugs and modifiable and genetic risk and protective factors associated with this process. We will evaluate the associations of mucus plugs with lung function, respiratory symptoms, and chronic bronchitis and examine whether 5-yr. persistent CT-based mucus plugs are associated with the decline in FEV1 and future COPD. Also, we will assess whether modifiable factors, including air pollution and marijuana smoking are associated with increased odds of CT-based mucus plugs and whether cardiorespiratory fitness is related in an opposing manner. Finally, we will determine genetic resilience/susceptibility to mucus pathology. We will use CT data from the FHS and CARDIA cohorts and genome-wide sequencing data from the TOPMed initiative to identify common and rare variants associated with CT-based mucus plugging.

**Ethics and dissemination:**

The Mass General Brigham Institutional Review Board approved the study. Findings will be disseminated through peer-reviewed journals and at professional conferences.

**Conclusion:**

Determine whether the presence of CT-based mucus plugs is associated with lung health impairment, including reduced FEV1, more respiratory symptoms, and asthma. Identify modifiable risk and protective factors, such as pollution, exercise, smoking, and fitness that are associated with mucus plugs.

## Introduction

Mucus pathology plays a critical role in airway diseases, such as Chronic Bronchitis (CB), asthma, and Chronic Obstructive Pulmonary Disease (COPD) [1–9]. COPD affects ~29 million people and is the 4^th^ leading cause of death in the US [10,11]. Mucus pathology and plug formation are complex processes in response to multiple inhaled noxious particles, including cigarette smoke and air pollution [1,12,13]. The public health and clinical relevance of airway mucus pathology are better understood through its clinical manifestations, including chronic cough and phlegm and chronic mucus hypersecretion/CB [14–19]. The prevalence of chronic respiratory symptoms in primary care is up to ~ 40% [20]. In a large study, 32% of ~98,000 community-living individuals with normal lung function reported chronic respiratory symptoms. More importantly, those symptoms were indicators of an increased risk of future hospitalization and mortality [14].

We and others have also demonstrated that in smokers with COPD, mucus plugs occluding the lumen of medium-to-large-sized airways (i.e., 2–10 mm lumen diameter), those visualizable on CT, are associated with impaired lung function, worse quality of life, and increased all-cause mortality [6,7,21]. We posit that CT assessment of airway mucus pathology applies to community-living individuals [22,23].

Extensive research has been conducted on chronic respiratory disorders. While this has brought new insights into diseases’ clinical variability and biological basis, attention has been limited to patients with established and even advanced disorders. Suppose clinical care is to identify early risk factors and prevent disease. In that case, new work is needed to identify those at high risk of future diseases, those who may have poor lung health but do not yet meet existing diagnostic criteria for an established airway disease [17]. We have shown that smokers have persistent mucus plugs on CT over a 5-year follow-up [6], but whether this pattern represents a risk factor for airway disease in community-living adults is unknown.

In this study, we will also determine the role of modifiable factors, such as air pollution, marijuana, and fitness, on mucus pathology, which may inform specific preventive and treatment strategies for lung health (e.g., air purification). Exposure to air pollution and cigarette smoke induce pathologic mucus production in the airways; however, not all individuals exposed to the same set of factors develop comparable mucus dysfunction and its consequences, suggesting genetic susceptibility or resilience may have a role [23].

Our team has implicated *TP53* gene variants in increased mucus synthesis and sustained the lifespan of metaplastic bronchial mucous cells. Further, smokers with the p53^Arg^ are at increased risk of CB (OR=1.59; p=0.03) [24].

We propose that CT-based mucus plugs are an intermediate phenotype that will enable us to investigate the clinical implications of mucus pathology and assess its contribution to the risk of airway disease. To test our hypothesis, we will use data from two population-based cohort studies: The Coronary Artery Risk Development in Young Adults (CARDIA) study and the Framingham Heart Study (FHS) [25–27]. The study protocol presented here was funded by the National Heart Lung and Blood Institute (Grant R01-HL164824).

### Specific aims

Our overall hypothesis is that CT-based mucus plugs are an intermediate phenotype that will enable us to examine the clinical implications of mucus pathology and gauge its contribution to airway disease risk in population-based studies. The study has three specific aims:

- Determine whether CT-based mucus plugs are associated with reduced FEV_1_, respiratory symptoms, and CB in community-living adults.
- Determine whether modifiable exposures (long-term air pollution, marijuana use, and fitness) are associated with CT-based mucus plugs.
- Assess the genetic resilience/susceptibility to mucus pathology by examining whether common and rare variants are associated with CT-based mucus plugging in community-living adults.

We will use existing imaging, clinical, environmental, and genetics data already collected from the CARDIA and FHS cohort, thus not exerting additional burden to the study participants and avoiding further radiation exposure.

## Materials and Methods

### Study design

The present study will use prospectively collected data from the CARDIA and FHS cohorts. CARDIA is a United States (US) National Institutes of Health (NIH)-funded population-based cohort of 5,115 young adults recruited from 1985 to 1986 to explore the evolution of cardiovascular disease risk factors [25]. Participants were examined at four sites across the US during enrollment. In-person examinations occurred at baseline (year (Y) 0), Y2, Y5, Y7, Y10, Y15, Y20, Y25, and Y30. Examinations collected demographic, anthropometric, clinical (e.g., symptoms and tobacco and marihuana smoking history), fitness, chest CT, and spirometry data in a standardized manner. CARDIA also took and kept blood samples. Participants’ blood samples were sent to the NIH Trans-Omics for Precision Medicine (TopMed) program to perform whole-genome sequencing. We will use chest CT images from the Y20 (n=3,122) and Y25 (n=3,498) examinations to score airway mucus plugs.

The FHS started in 1948 as a prospective investigation of cardiovascular disease, and we will use participants from the FHS Multidetector Computed Tomography 2 (MDCT2) sub-study (n=2750). Participants of the MDCT2 sub-study are from the FHS Offspring Cohort (1971) and the FHS Third Generation (2002) cohort [27]. The FHS third generation participants are the children of the offspring’s. FHS also collected demographic, anthropometric, clinical (e.g., symptoms and tobacco smoking history), air pollution, spirometry, and chest CT data in a standardized manner. FHS participants’ blood samples were also sent to the NIH TopMed program to perform whole-genome sequencing. We will use chest CT images from the MDCT2 sub-study to identify and score airway mucus plugs. Characteristics of the Y20 CARDIA and FHS participants are depicted in Table 1.

### Patient and public involvement statement

Patients and/or the public were not involved in the design, conduct, reporting or dissemination plans of this research.

### Eligibility

In the CARDIA cohort, eligibility was limited to young adults aged (18–30) years in 1985 to 1986 with no evidence of long-term diseases or disabilities. In the FHS cohort, eligibility was limited to participants aged 20 years or older and had at least one parent who was a member of the Offspring Cohort [27]. All individuals known to be eligible for the inclusion in the Third Generation Cohort were sent invitation letters along with response letters, and high interest was indicated by return of response cards, which led to more participation than anticipated.

### Participant background

At Y20, the mean age was 45 years; 38% were smokers, and 9%-19% reported cough, phlegm, or dyspnea. CARDIA included a balanced number of male and female and Black and White participants, whereas FHS had well-balanced sexes (M/F) but only included White participants. In the FHS-MDCT2 cohort, the mean age was 59 years; 51% were former and current smokers, and 7%-11% reported respiratory symptoms. Participants of both population-based cohort studies include varying cardiorespiratory fitness, air pollution exposure, and tobacco and marijuana smoking histories.

### Study data collection

The CARDIA and FHS studies used standardized questionnaires and procedures to collect imaging, clinical (e.g., marijuana history), environmental, fitness, and genetic data. Also, the methodology to assess mucus plugs on CT scans for this study is detailed below.

The Institutional Review Board of Mass General Brigham approved this study. All participants provided written consent.

### Chest CT scans

All participants underwent chest CT scanning. CARDIA patients underwent a noncontrast electrocardiography-gated chest CT scan reconstructed at 2.5 mm to 3 mm slice thickness [28]. FHS-MDCT2 sub-study patients underwent lung volumetric CT scans in the supine position, reconstructed by a sharp lung algorithm with a 0.625 mm thickness and a 50 cm field of view [29]. Although there are differences in reconstructions between cohorts, the quality of CT scans reconstruction parameters is adequate for this study to survey the airways.

### Defining mucus plugs

**Figure 1:**
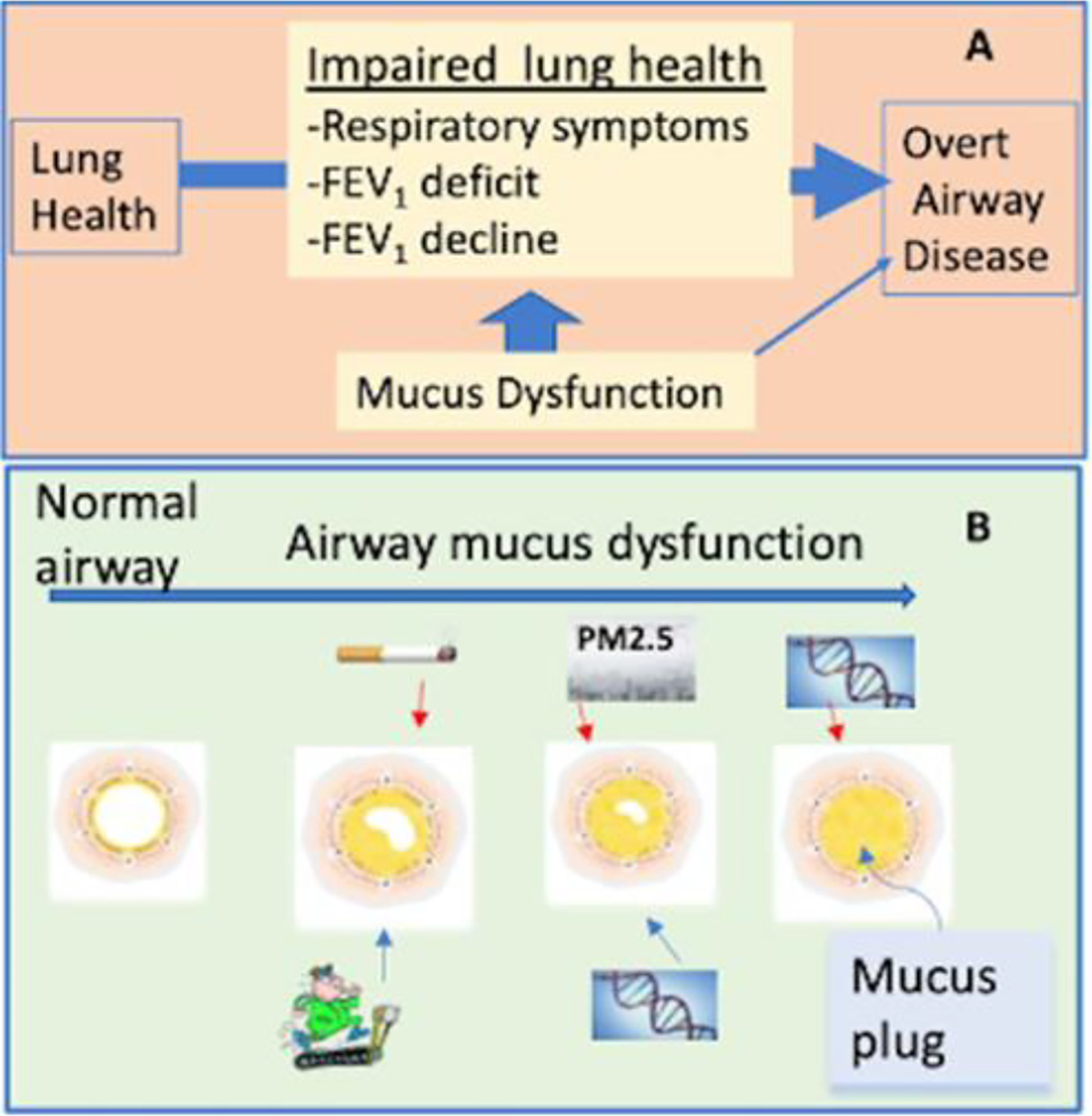
A) **Progression from healthy to overt airway disease:** Impaired lung health is between these extremes. Mucus dysfunction is associated with impaired lung health through multiple factors yet provides target to intercept pulmonary disease before they become chronic; B) **Airway mucus dysfunction:** Tobacco, marijuana, and PM_2.5_ increases mucus plugs whereas fitness can protect against plug formation. Genetic variants resilience or susceptibility may also play a role in airway mucus biology.

A mucus plug is defined as an opacity that completely occludes the lumen of an airway on sequential slices, regardless of the generation and orientation of the bronchus (Figure 1) [3,4]. The opacity can be round or cylindrical in shape and found in one or many airway branches. Plugging is identified in medium-to-large-sized airways on CT.

### Mucus plug score

**Figure 2:**
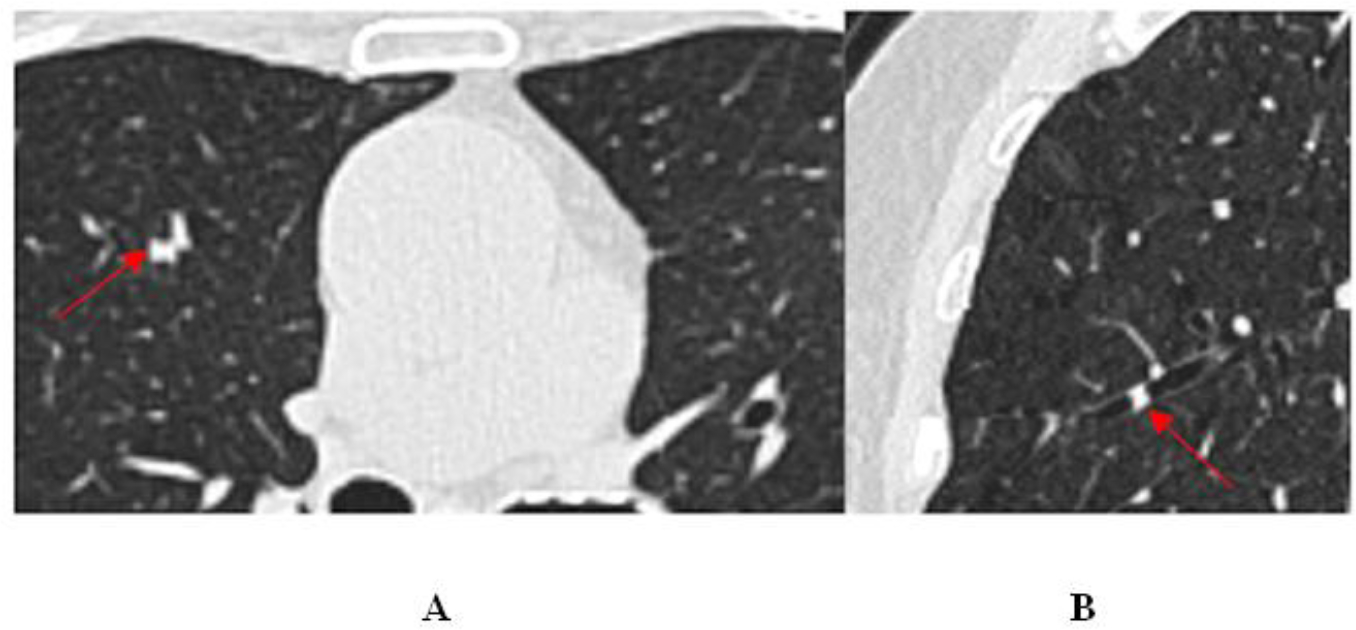
A) Axial CT image showing a mucus plug occluding an airway (arrow) in the right upper lobe; B) The same mucus plug in the sagittal plane.

The scoring method was developed based on the number of pulmonary segments with mucus plugs regardless of the orientation of the bronchus (Figure 2). For instance, a mucus plug identified in the lower right lobe’s posterior and lateral basal segments yields a score of two. The score will range from 0 to 18, where 0 means no plugging and higher numbers indicate a greater extent of plugs.

### Reading method

The readers who will score mucus plugs comprise thoracic radiologists, a pulmonologist, physicians, and graduate students. Participating readers undergo training, which consisted of identifying a variety of mucus plugs on a set of typical CT images, scoring a training set of CT scans to learn the scoring system, and scoring a sample set of 20 CT scans to verify the inter-reader agreement. The concordance correlation for inter-reader agreement of mucus plug score previously reported was 0.84 (95% Cl: 0.81–0.88) [6]. The Applied Chest Imaging Laboratory also has developed an automated method to detect mucus plugs, providing an alternate way to assess airway mucus pathology on CT images.

### Spirometry

Spirometry protocols were designed following the American Thoracic Society (ATS)/European Respiratory Society (ERS) guidelines. While CARDIA protocols did not include the use of a bronchodilator, the FHS-Offspring cohort (i.e., at examination 9) performed postbronchodilator measurements in those with airflow limitation. Given the epidemiological nature of this study, we will use prebronchodilator measures of lung function. Airflow limitation is defined as prebronchodilator Forced Expiratory Volume in 1 second (FEV_1_)/Forced Vital Capacity (FVC) ratio <0.70 or less than the lower limit of normal [30].

### Symptoms

Chronic respiratory symptoms include asthma, airway wall thickening, dyspnea, phlegm, emphysema, and bronchiectasis. CT-based mucus plugging is associated with dyspnea, phlegm, cough, and CB.

### Environmental exposures + Air pollution

Information on FHS participants’ residential address, PM_2.5_ levels, and proximity to a major road in relation to air quality was determined with a geospatial model. Models contained daily air pollutants, weather, and chemical exposures that integrate satellite-based aerosol measurements, Goddard Earth Observing System atmospheric, weather data, and local land data. Machine learning techniques were employed to refine the model based on ground-level measured exposure data to estimate participant daily exposures at high resolution with excellent out-of-sample cross-validation performance. Proximity to major roads was calculated *via* the U.S. Census Feature Class A1, A2, and A3 data [31–33].

### Marijuana exposure

Information on CARDIA participants’ marijuana smoking habits was collected from Y0 to Y25. Lifetime marijuana use was defined as the number of joints smoked from Y0 onwards, including Y20 and Y25 [34].

### Cardiorespiratory fitness

Participants had a symptom-limited, graded treadmill exercise test to measure cardiorespiratory fitness during the Y20 CARDIA exam. The modified Balke protocol consists of nine 2-minute stages of increasing difficulty in both speed and grade to a maximum of 5.6 mph at 25% grade (19.0 metabolic equivalent tasks). The test duration and maximal metabolic equivalent task were recorded [28,35].

### Genetics

NIH TOPMed whole-genome sequencing will be used on both cohorts to identify common and rare variants associated with CT mucus plugging [36]. The NIH TOPMed Study performed alignment and variant calling using a standardized pipeline detailed at nhlbiwgs. org, yielding high-quality whole genome sequencing variant calls.

### Hypothesis

There are three primary Hypothesis,

**Hypothesis 1:** CT-based mucus plugging is associated cross-sectionally with lower FEV_1_, higher burden of respiratory symptoms, and CB; persistent mucus plugs over time are associated with a decline in FEV_1_ and future development of COPD.

**Hypothesis 2:** Air pollution and lifetime marijuana use are associated with increased odds of CT-based mucus plugging, whereas cardiorespiratory fitness is protective against airway mucus plug formation.

**Hypothesis 3:** Common and rare variants are associated with CT-based mucus plugging.

### Analysis plan

To evaluate Hypothesis 1, we will conduct separate analyses for FHS and CARDIA. In CARDIA, linear and logistic multivariable models will be used to assess the cross-sectional relationships between CT-based mucus plugging and FEV_1_ % predicted (continuous), cough, phlegm, dyspnea, and bronchitis episodes (all binary). We will consider the following covariates: Age, sex, race, center, education level, BMI, ever-smoking status, pack-year, and history of asthma. In FHS, linear mixed-effect models will be used for FEV_1_ and generalized mixed-effect models for binary outcomes: Cough, phlegm, dyspnea, and CB. In these models, fixed effects, except for race, will be like those used in CARDIA. Since FHS includes participants with familial relatedness (parents and children), we will include a random effect to account for familial aggregation. Finally, we will conduct sex-stratified analyses for these outcomes, as preliminary studies suggest women have higher odds of mucus plugging.

We will use the CARDIA cohort to assess the association between CT-based mucus plugs and the outcome change in FEV_1_ over time, using Y20 and Y25 CT data. For these longitudinal analyses, we will classify changes in mucus plugging into two groups: Persistent Positive (mucus plugs at both Y20 and Y25) + Newly Formed (mucus plugs at Y25 but not at Y20) *vs*. Persistent Negative (no mucus plugs at both Y20 and Y25) + Resolved (mucus plugs at Y20 but not at Y25). The two latter groups combined are the referent group. We will use linear mixed models and generalized mixed models to assess whether that binary variable is associated with the outcomes decline in FEV_1_ and COPD at the Y30 exam. Decline in FEV_1_ will be defined as Y30 minus Y20 divided by 10, yielding changes per year. Unfortunately, CARDIA has no spirometry data at Y25. COPD is defined as a pre-bronchodilator FEV_1_/FVC ratio <0.7, an acceptable approach for epidemiologic studies [37,38]. The cutoff of 0.7 has been validated in population-based studies [39]. Models for FEV_1_ decline will include age, sex, race, center, education level, BMI, ever-smoking status, pack-year, baseline FEV_1_, and history of asthma. Time-variant variables include pack-year, smoking status, and BMI. Also, we will perform analyses to explore sex and race differences in the associations of interest, drawing from the sex and racial-balanced number of CARDIA participants.

## Results and Discussion

### Power calculation and sample size for hypothesis 1

Detectable effect sizes are calculated for FEV_1_ decline and future COPD to reach at least 80% power, assuming a sample size of 2,600 participants with CARDIA Y20 and Y25 CT data and an alpha value of 0.05. If the prevalence of persistent mucus plugging is 10%, we detect a 53 mL FEV_1_ decline and a 1.87 odds ratio of future COPD. With a prevalence of persistent mucus plugging of 20%, we detect a 40 mL FEV_1_ decline and an odds ratio of 1.61 for future COPD.

To evaluate Hypothesis 2, we will perform separate analyses in CARDIA and FHS. For CARDIA, we will use a logistic model to assess the associated between lifetime marijuana smoking and the outcome CT-based mucus plugging (i.e., binary variable defined as mucus plug score 0 *vs*. >0). Based on prior studies, marijuana will be categorized to indicate high and low consumption. We will consider adjustments for age, race, and education attained, BMI, FEV_1_, presence of emphysema on CT, and asthma history. Also, we will build separate models to examine the relationships between tobacco smoking (ever *vs*. never, pack-years) and mucus plugging and compared them. Finally, we will explore whether tobacco and marijuana smoking have independent or additive effects on mucus plug formation. In separate logistic models, we will test the association between fitness (i.e., treadmill duration) and mucus plugging. Further, we think that fitness may counter the effect of tobacco smoking on mucus formation. Thus, in secondary analyses, we will test whether fitness modifies (i.e., mitigates) the impact of cigarette smoking status and pack-years on CT-based mucus plugging using the appropriate interactions terms. For FHS, a generalized mixed model will test the association between air pollution and distance to roadways (exposures) with CT-based mucus plugging as the outcome. Based on previous studies, we will consider using two exposures: 5-year PM_2.5_ average concentration (2004–2008) before CT scanning (2008–2011) and distance to major roads as categories. We will consider the following covariates: Age, sex, education level, BMI, smoking (ever *vs*. never), pack-years, the value of owner-occupied housing, population density in the census track, FEV_1_, and CT measures of emphysema and airway-wall thickening. A random effect will be used to account for FHS familial aggregation.

### Power calculation and sample size for hypothesis 2

Detectable effect sizes are also computed on the risk of mucus plugging (binary outcome) for fitness and PM_2.5_ and reach at least 80% power, assuming an alpha level of 0.05 and sample sizes of 2,677 (fitness, CARDIA) and 2,340 (PM_2.5_, FHS). CARDIA and FHS sample sizes were smaller due to missing fitness and PM_2.5_ data. In a prevalence of CT-based mucus plugs of 5%, there is a 1.29 relative risk of mucus plugging per 1 SD decrease in fitness and a 1.31 relative risk per 1 SD increase in PM_2.5_ mcg/m^3^. In a prevalence of CT-based mucus plugs of 20%, there is a 1.15 relative risk of mucus plugging per 1 SD decrease in fitness and a 1.16 relative risk per 1 SD increase in PM_2.5_ mcg/m^3^.

To evaluate Hypothesis 3, we will use NIH TOPMed data for common and rare variants. For common variants, we will examine variants with minor allele counts of above 30 in FHS and CARDIA. We will perform a combined analysis, leveraging the single dataset available for all TOPMed [40]. The main analysis will include all participants and adjust for age, sex, current smoking, pack-years, sequencing center/study, race, familial aggregation, and principal ancestry components in a linear mixed model implemented in SAIGE [41].To balance maximum power with the desire for replication, we will apply a tiered strategy previously used and consider ‘Tier 1’ signals those that reach pre-determined levels in FHS and replicate in CARDIA, and ‘Tier 2’ signals those that reach significance in the analysis of FHS and CARDIA, and that have nominally significant P values in both studies.

Given the importance of cigarette smoking and potential air pollution in mucus plug formation, we will also perform secondary analysis using common variants including both the main effect and a gene by environment interaction using a two-degree of freedom joint test that improves power in the setting of interaction [42]. Due to the differences in prevalence among races, we will also examine results by ancestry and perform admixture analysis in Black participants in CARDIA using LAMP-LD, as we have previously performed [43].

For rare variants, aggregating variants can increase power. We will use the recently developed ACAT-O (Aggregated Cauchy Association Test, Omnibus), which combines set-based tests including burden, SKAT, and ACAT-V, improves power in the setting of sparsity, and is superior to earlier methods such as SKAT-O [44]. We will test aggregating by gene or by sliding window region and also examine the effects of limiting our analysis to coding variants (nonsynonymous, stop, or splice) or those with putative regulatory function (e.g., based on overlap with open chromatin in lung-related cells) [45].

We also anticipate examining a specific set of ~100 genes known to be important for mucus dysfunction (e.g., *TP53*) and muco-obstructive diseases such as COPD and bronchiectasis (e.g., *FAM13A, MUC5B, MUC5AC, CFTR,* and *DNAH5*). This secondary analysis decreases the multiple comparison penalty from genome-wide approaches while enabling a more detailed examination of genes with a higher likelihood of association. As a secondary analysis, we will perform a genome-wide association.

We will analyze heritability, genetic correlation, functional enrichment, and potential causal genes to analyze and interpret our genetic association results. We will use GCTA-SC and GCTA-LDMS in our European sample to estimate heritability and genetic correlation between this CT phenotype and lung function [46]. To identify potential causal genes, we will rely on multiple approaches, including expression Quantitative Trait Loci (eQTL) in lung and blood, examination of *DNAase* hypersensitivity sites, chromosome conformation (Hi-C), and gene set and pathway methods (depict), as previously performed [47].

### Power calculation and sample size for hypothesis 3

When assessing genetic variance to CT-based airway mucus plugs, in a total sample size of 5,427 (FHS+Y20 CARDIA) and assuming a prevalence of mucus plugging of 10%, we have ~80% power to identify a variant at a P<1 x 10^−4^ with an allele frequency of 40% and an odds ratio of ~1.3. In addition, we have >80% power at alpha=5 x 10^−8^ in our genome-wide analysis to identify a variant that increases risk by 1.45.

## Conclusion

Determine whether the presence of CT-based mucus plugs is associated with lung health impairment, including reduced FEV_1_, more respiratory symptoms, and asthma. Identify modifiable risk and protective factors, such as pollution, exercise, smoking, and fitness that are associated with mucus plugs. Determine genetic variants associated with susceptibility or resilience to mucus pathology as measured on CT. Obtain insight into mucus plugs as a potential target for preventive and therapeutic strategies and inform policy for mitigation of mucus pathology. Understand the clinical implications of mucus pathology and its association in the progression of airway disease.

### Strengths and Limitations of this study

1) Utilization of data from two well-characterized large community-based US cohorts, 2) Use of chest CT scans to identify and quantify mucus plugs, providing a more objective and reproducible measure of airway pathology, 3) Use of whole-genome sequencing to identify common and rare genetic variants associated with mucus pathology, 4) Only the inclusion of participants self-identified as non-Hispanic white and non-Hispanic black, 5) A limitation of retrospective study design using prospectively collected data.

### Ethics and Dissemination

The present study was approved by the Mass General Brigham Institutional Review Board (2022P000723). Participants were recruited voluntarily for these studies. Written informed consent was obtained for CT scanning, clinical data, blood sample collection, and genomic analyses. Participants were under no obligation to participate. A potential risk is the risk of participant de-identification or the release of personal health information. We will use genotype and phenotype files, which are only identified by study ID numbers; the investigators are not members of the Data Coordinating Center of these studies and will therefore not have access to any information that is not already de-identified. To protect against inadvertent disclosure of identity, we will make all study data available to study investigators using the de-identified study identifications assigned by the CARDIA and FHS studies. The links to identifiers are not available to the investigators. All study data is stored on a secured, password-protected computer system at Brigham and Women’s Hospital (BWH) and CARDIA and FHS Data Coordinating Centres.

We plan to disseminate the findings of this study through peer-reviewed journals and professional scientific conferences, such as the American Thoracic Society’s annual Conference.

## Figures and Tables

**Table 1:** Characteristics of 20 years CARDIA and FHS participants.

Characteristic	CARDIA (n=3,122)	FHS (n=2,750)
Age, years	45 (4)	59 (12)
Female sex*	57%	50%
White race*	53%	100%
Education status		
High school or less	23%	23%
More than high school	28%	31%
College degree or more	50%	46%
BMI, kg/m^2^	30.2 (7.2)	28.5 (5.4)
Tobacco smoking status		
Never	62%	48%
Current	18%	6%
Former	20%	45%
Pack-years smoked	5.1 (9.4)	9.6 (16.1)
Lifetime marihuana smoking, No. of joints/years	366/20 yrs.	-
PM_2.5_, mcg/m^3^	-	8.9(1.5)
Fitness (treadmill duration), min	7.1 (2.7)	-
Cough	9%	7%
Phlegm	9%	6%
Dyspnea	19%	11%
Asthma	13%	6%
Episodes of bronchitis ^/CB	4%	7%
Spirometric COPD	8%	21%
FEV1 % predicted	92 (16)	98 (15)
FVC % predicted	94 (15)	102 (13)
FEV_1_/FVC	0.77 (0.1)	0.74 (0.1)
Emphysema&/LAA-950, % on CT	2.4%	26.9 (7.2)
Airway wall thickness, mm	-	1.1 (0.1)

**Note:** Data are presented as mean (standard deviation) and proportion, %: Percentages were calculated with data available for each variable;

* : Race and sex data are from Y25 exam;

^ : CB questions are available for FHS only;

Emphysema (yes/no) was visually assessed (CARDIA); LAA: % of lung attenuation areas under -950 Hounsfield units; Cells with -means no data.

## References

[1] Boucher RC (2019) Muco-obstructive lung diseases. N Engl J Med 381:1941–1953. 10.1056/NEJMra1813799 31091375

[2] Fahy JV, Dickey BF (2010) Airway mucus function and dysfunction. N Engl J Med 363:2233–2247. 21121836 10.1056/NEJMra0910061 PMC4048736

[3] Svenningsen S, Haider E, Boylan C, Mukherjee M, Eddy RL, (2019) CT and functional MRI to evaluate airway mucus in severe asthma. Chest 155:1178–1189. 30910637 10.1016/j.chest.2019.02.403

[4] Dunican EM, Elicker BM, Gierada DS, Nagle SK, Schiebler ML, (2018) Mucus plugs in patients with asthma linked to eosinophilia and airflow obstruction. J Clin Invest 128:997–1009. 29400693 10.1172/JCI95693 PMC5824874

[5] Chassagnon GP, Burgelm R (2021) Mucus plugs in medium-sized airways: A novel imaging biomarker for phenotyping Chronic Obstructive Pulmonary Disease. Am J Respir Crit Care Med 203:932–934. 33264069 10.1164/rccm.202011-4178ED PMC8048760

[6] Okajima Y, Come CE, Nardelli P, Sonavane SK, Yenet A, (2020) Luminal plugging on chest CT Scan: Association with lung function, Quality of Life, and COPD clinical phenotypes. Chest 158:121–130. 32017932 10.1016/j.chest.2019.12.046 PMC7339234

[7] Dunican EM, Elicker BM, Henry T, Gierada DS, Schiebler ML, (2021) Mucus plugs and emphysema in the pathophysiology of airflow obstruction and hypoxemia in smokers. Am J Respir Crit Care Med 203:957–968. 33180550 10.1164/rccm.202006-2248OC PMC8048745

[8] Kim V, Dolliver WR, Nath HP, Grumley SA, Terry N, (2021) Mucus plugging on computed tomography and chronic bronchitis in chronic obstructive pulmonary disease. Respir Res 22:110. 33865371 10.1186/s12931-021-01712-0 PMC8053272

[9] MettlerSK, NathHP, GrumleyS, OrejasJL, DolliveretWL, (2023) Silent airway mucus plugs in COPD and clinical implications. Chest 3692:05825–05827.10.1016/j.chest.2023.11.03338013161

[10] Ford ES, Mannino DM, Wheaton AG, Giles WH, Cantrell LP, (2013) Trends in the prevalence of obstructive and restrictive lung function among adults in the United States: Findings from the National Health and Nutrition Examination surveys from 1988–1994 to 2007–2010. Chest 143:1395–1406. 23715520 10.1378/chest.12-1135 PMC4563801

[11] Ma J, Ward EM, Siegel RL, Jema A (2015) Temporal trends in mortality in the United States, 1969–2013. Jama 314:1731–1739. 26505597 10.1001/jama.2015.12319

[12] Button B, Goodell HP, Atieh E, Chen YC, Williams R, (2018) Roles of mucus adhesion and cohesion in cough clearance. Proc Natl Acad Sci U S A 115:12501–12506. 30420506 10.1073/pnas.1811787115 PMC6298066

[13] Kesimer M, Ford AA, Ceppe A, Radicioni G, Cao R, (2017) Airway mucin concentration as a marker of chronic bronchitis. N Engl J Med 377:911–922. 28877023 10.1056/NEJMoa1701632 PMC5706541

[14] Çolak Y, Nordestgaard BG, Vestbo J, Lange P, Afzal S (2019) Prognostic significance of chronic respiratory symptoms in individuals with normal spirometry. Eur Respir J 54:1900734. 31248954 10.1183/13993003.00734-2019

[15] Allinson JP, Hardy R, Donaldson GC, Shaheen SO, Kuh D, (2016) The presence of chronic mucus hypersecretion across adult life in relation to chronic obstructive pulmonary disease development. Am J Respir Crit Care Med 193:662–672. 26695373 10.1164/rccm.201511-2210OC PMC4824943

[16] Guerra S, Sherrill DL, Venker C, Ceccato CM, Halonen M, (2009) Chronic bronchitis before age 50 years predicts incident airflow limitation and mortality risk. Thorax 64:894–900. 19581277 10.1136/thx.2008.110619 PMC4706745

[17] Puhan MA (2019) Chronic respiratory symptoms but normal lung function: Substantial disease burden but little evidence to inform practice. Eur Respiratory Soc 19:1901363. 10.1183/13993003.01363-2019 31537654

[18] Mejza F, Gnatiuc L, Buist AS, Vollmer WM, Lamprecht B, (2017) Prevalence and burden of chronic bronchitis symptoms: Results from the BOLD study. Eur Respir J 50:1700621. 29167298 10.1183/13993003.00621-2017 PMC5699921

[19] Woodruff PG, Barr RG, Bleecker E, Christenson SA, Couper D, (2016) Clinical significance of symptoms in smokers with preserved pulmonary function. N Engl J Med 374:1811–1821. 27168432 10.1056/NEJMoa1505971 PMC4968204

[20] Hasselgren M, Arne M, Lindahl A, Janson S, Lundbäck B (2001) Estimated prevalences of respiratory symptoms, asthma and chronic obstructive pulmonary disease related to detection rate in primary health care. Scand J Prim Health Care 19:54–57. 11303549 10.1080/028134301300034701

[21] Diaz AA, Orejas JL, Grumley S, Nath HP, Wang W, (2023) Airway-Occluding Mucus plugs and mortality in patients with Chronic Obstructive Pulmonary Disease. JAMA 329:1832–1839. 37210745 10.1001/jama.2023.2065 PMC10201404

[22] Liu GY, Kalhan R (2021) Impaired respiratory health and life course transitions from health to chronic lung disease. Chest 160:879–889. 33865834 10.1016/j.chest.2021.04.009 PMC8449011

[23] Silverman EK, Chapman HA, Drazen JM, Weiss ST, Rosner B, (1998) Genetic epidemiology of severe, early-onset chronic obstructive pulmonary disease: Risk to relatives for airflow obstruction and chronic bronchitis. Am J Respir Crit Care Med 157:1770–1778. 9620904 10.1164/ajrccm.157.6.9706014

[24] Chand HS, Montano G, Huang X, Randell SH, Mebratu Y, (2014) A genetic variant of p53 restricts the mucous secretory phenotype by regulating SPDEF and Bcl-2 expression. Nat Commun 5:5567. 25429397 10.1038/ncomms6567 PMC4247165

[25] Friedman GD, Cutter GR, Donahue RP, Hughes GH, Hulley SB, (1988) CARDIA: Study design, recruitment, and some characteristics of the examined subjects. J Clin Epidemiol 41:1105–1116. 3204420 10.1016/0895-4356(88)90080-7

[26] Kannel WB, Feinleib M, McNamara PM, Garrison RJ, Castelli WP (1979) An investigation of coronary heart disease in families: The Framingham Offspring Study. Am J Epidemiol 110:281–290. 474565 10.1093/oxfordjournals.aje.a112813

[27] Splansky GL, Corey D, Yang Q, Atwood LD, Cupples LA, (2007) The third generation cohort of the National Heart, Lung, and Blood Institute’s Framingham Heart Study: Design, recruitment, and initial examination. Am J Epidemiol 165:1328–1335. 17372189 10.1093/aje/kwm021

[28] Diaz AA, Colangelo LA, Okajima Y, Yen A, Sala MA, (2021) Association between cardiorespiratory fitness and bronchiectasis at CT: A long-term population-based study of healthy young adults aged 18–30 years in the CARDIA study. Radiology 300:190–196. 33904771 10.1148/radiol.2021203874 PMC8248949

[29] Rice MB, Li W, Dorans KS, Wilker EH, Ljungman P, (2018) Exposure to traffic emissions and fine particulate matter and computed tomography measures of the lung and airways. Epidemiology 29:333–341. 29384790 10.1097/EDE.0000000000000809 PMC6095201

[30] Oelsner EC, Balte PP, Cassano PA, Couper D, Enright PL, (2018) Harmonization of respiratory data from 9 US population-based cohorts: The NHLBI Pooled Cohorts Study. Am J Epidemiol 187:2265–2278. 29982273 10.1093/aje/kwy139 PMC6211239

[31] Rice MB, Ljungman PL, Wilker EH, Dorans KS, Gold DR, (2015) Long-term exposure to traffic emissions and fine particulate matter and lung function decline in the Framingham heart study. Am J Respir Crit Care Med 191:656–664. 25590631 10.1164/rccm.201410-1875OC PMC4384780

[32] Synn AJ, Byanova KL, Li W, Gold DR, Di Q, (2021) Ambient air pollution exposure and radiographic pulmonary vascular volumes. Environ Epidemiol 5:e143. 33870015 10.1097/EE9.0000000000000143 PMC8043731

[33] Rice MB, Li W, Schwartz J, Di Q, Kloog I, (2019) Ambient air pollution exposure and risk and progression of interstitial lung abnormalities: The Framingham Heart Study. Thorax 74:1063–1069. 31391318 10.1136/thoraxjnl-2018-212877 PMC7136036

[34] Pletcher MJ, Vittinghoff E, Kalhan R, Richman J, Safford M, (2012) Association between marijuana exposure and pulmonary function over 20 years. JAMA 307:173–181. 22235088 10.1001/jama.2011.1961 PMC3840897

[35] Benck LR, Cuttica MJ, Colangelo LA, Sidney S, Dransfield MT, (2017) Association between cardiorespiratory fitness and lung health from young adulthood to middle age. Am J Respir Crit Care Med 195:1236–1243. 28248551 10.1164/rccm.201610-2089OC PMC5439017

[36] Prokopenko D, Sakornsakolpat P, Fier HL, Qiao D, Parker MM, (2018) Whole-genome sequencing in severe chronic obstructive pulmonary disease. Am J Respir Cell Mol Biol 59:614–622. 29949718 10.1165/rcmb.2018-0088OC PMC6236690

[37] Khalid F, Wang W, Mannino D, Diaz AA (2022) Prevalence and population attributable risk for early chronic obstructive pulmonary disease in US Hispanic/Latino individuals. Ann Am Thorac Soc 19:363–371. 34530700 10.1513/AnnalsATS.202103-253OC PMC8937229

[38] Çolak Y, Afzal S, Nordestgaard BG, Vestbo J, Lange P (2020) Prevalence, characteristics, and prognosis of early chronic obstructive pulmonary disease. The Copenhagen General Population Study. Am J Respir Crit Care Med 15;201:671–680. 31770495 10.1164/rccm.201908-1644OC PMC7068820

[39] Bhatt SP, Balte PP, Schwartz JE, Cassano PA, Couper D, (2019) Discriminative accuracy of FEV_1_: FVC thresholds for COPD-related hospitalization and mortality. JAMA 321:2438–2447. 31237643 10.1001/jama.2019.7233 PMC6593636

[40] Hobbs BD, de Jong K, Lamontagne M, Bossé Y, Shrine N, (2017) Genetic loci associated with chronic obstructive pulmonary disease overlap with loci for lung function and pulmonary fibrosis. Nat Genet 49:426–432. 28166215 10.1038/ng.3752 PMC5381275

[41] Zhou W, Nielsen JB, Fritsche LG, Dey R, Gabrielsen ME, (2018) Efficiently controlling for case-control imbalance and sample relatedness in large-scale genetic association studies. Nat Genet 50:1335–1341. 30104761 10.1038/s41588-018-0184-y PMC6119127

[42] Manning AK, LaValley M, Liu CT, Rice K, An P, (2011) Meta‐analysis of gene‐environment interaction: Joint estimation of SNP and SNP × environment regression coefficients. Genet Epidemiol 35:11–18. 21181894 10.1002/gepi.20546 PMC3312394

[43] Parker MM, Foreman MG, Abel HJ, Mathias RA, Hetmanski JB, (2014) Admixture mapping identifies a quantitative trait locus associated with FEV_1_/FVC in the COPDGene Study. Genet Epidemiol 38:652–659. 25112515 10.1002/gepi.21847 PMC4190160

[44] Liu Y, Chen S, Li Z, Morrison AC, Boerwinkle E, (2019) ACAT: A fast and powerful p value combination method for rare-variant analysis in sequencing studies. Am J Hum Genet 104:410–421. 30849328 10.1016/j.ajhg.2019.01.002 PMC6407498

[45] Prokopenko D, Sakornsakolpat P, Fier HL, Qiao D, Parker MM, (2018) Whole-genome sequencing in severe chronic obstructive pulmonary disease. Am J Respir Cell Mol Biol 59:614–622. 29949718 10.1165/rcmb.2018-0088OC PMC6236690

[46] Yang J, Bakshi A, Zhu Z, Hemani G, Vinkhuyzen AA, (2015) Genetic variance estimation with imputed variants finds negligible missing heritability for human height and body mass index. Nat Genet 47:1114–1120. 26323059 10.1038/ng.3390 PMC4589513

[47] Sakornsakolpat P, Prokopenko D, Lamontagne M, Reeve NF, Guyatt AL, (2019) Genetic landscape of chronic obstructive pulmonary disease identifies heterogeneous cell-type and phenotype associations. Nat Genet 51:494–505. 30804561 10.1038/s41588-018-0342-2 PMC6546635

